# Screening for Familial *APP* Mutations in Sporadic Cerebral Amyloid Angiopathy

**DOI:** 10.1371/journal.pone.0013949

**Published:** 2010-11-11

**Authors:** Alessandro Biffi, Anna Plourde, Yiping Shen, Robert Onofrio, Eric E. Smith, Matthew Frosch, Claudia M. Prada, James Gusella, Steven M. Greenberg, Jonathan Rosand

**Affiliations:** 1 Center for Human Genetic Research, Massachusetts General Hospital, Boston, Massachusetts, United States of America; 2 Hemorrhagic Stroke Research Group, Massachusetts General Hospital, Boston, Massachusetts, United States of America; 3 Program in Medical and Population Genetics, Broad Institute of MIT and Harvard, Cambridge, Massachusetts, United States of America; 4 Department of Clinical Neurosciences, University of Calgary, Calgary, Alberta, Canada; 5 Massachusetts General Hospital Institute for Neurodegenerative Disease, Massachusetts General Hospital, Boston, Massachusetts, United States of America; National University of Singapore, Singapore

## Abstract

**Background:**

Advances in genetic technology have revealed that variation in the same gene can cause both rare familial and common sporadic forms of the same disease. Cerebral amyloid angiopathy (CAA), a common cause of symptomatic intracerebral hemorrhage (ICH) in the elderly, can also occur in families in an autosomal dominant pattern. The majority of affected families harbor mutations in the Beta amyloid Peptide (Aβ) coding region of the gene for amyloid precursor protein (*APP*) or have duplications of chromosomal segments containing *APP*.

**Methodology/Principal Findings:**

A total of 58 subjects with a diagnosis of probable or definite CAA according to validated criteria were included in the present study. We sequenced the Aβ coding region of *APP* in 58 individuals and performed multiplex ligation-dependent probe amplification to determine *APP* gene dosage in 60. No patient harbored a known or novel *APP* mutation or gene duplication. The frequency of mutations investigated in the present study is estimated to range from 0% to 8% in individuals with probable CAA in the general population, based on the ascertained sample size.

**Conclusions/Significance:**

We found no evidence that variants at loci associated with familial CAA play a role in sporadic CAA. Based on our findings, these rare highly-penetrant mutations are unlikely to be seen in sporadic CAA patients. Therefore, our results do not support systematic genetic screening of CAA patients who lack a strong family history of hemorrhage or dementia.

## Introduction

Recent advances in genetic technology have clarified that variation in the same gene can cause both familial (usually more severe and/or earlier onset) and sporadic forms of the same disease. For example, rare highly penetrant sequence variants in several genes (*HNF4A, GCK, TCF1/HNF1A, TCF2/HNF1B*) invariably cause a monogenic disorder known as maturity-onset diabetes of the young (MODY), while common less penetrant variants in the same genes are risk factors for multifactorial type 2 diabetes. [1] Similarly, rare and common sequence variants within the ABCC8 gene have been associated with monogenic disorders (congenital hyperinsulinism of infancy), as well as sporadic type 2 diabetes. [2], [3]

The effect of more common variants is generally much less potent than that of rare variants, and as a result, for example, not all carriers of *ABCC8* variants develop diabetes. However, clustering of common and rare disease-causing variants in a single gene provides additional insight into pathophysiology, and may prove crucial for individual risk prediction.

Cerebral amyloid angiopathy (CAA) is characterized by β-amyloid peptide (Aβ) deposition and destruction of the vessel walls of capillaries, arterioles and small- and medium-sized arteries of the cerebral cortex, leptomeninges and cerebellum. In its sporadic form CAA is a common cause of intracerebral hemorrhage (ICH), [4] MRI-detected white matter hyperintensity (WMH) and cognitive dysfunction. [5], [6] Vascular amyloid, like the amyloid plaques in Alzheimer disease (AD), is composed chiefly of a 39- to 43- amino acid proteolytic fragment of the β-amyloid precursor protein (APP). A large body of data links the ε2 and ε4 alleles of APOE to CAA susceptibility. [7] Epidemiological data, however, point to an even larger role for genetic variation as a risk factor for CAA, suggesting that there are other loci in the genome that modulate risk. [8]

In addition to occurring as a spontaneous condition in the elderly, CAA also manifests as a rare familial syndrome in which manifestations generally develop earlier in life. Most familial forms of CAA involve mutations within *APP.* [8]–[14], and present with vascular deposition of Aβ causing a clinical phenotype akin to sporadic CAA, including either ICH or dementia as the primary clinical features. Besides the easily identifiable family inheritance pattern, familial CAA can be differentiated from sporadic CAA because of the average earlier age of onset (<55 years) and greater severity. All known *APP* mutations associated with CAA cluster within the Aβ-coding region of the gene (exons 16 and 17). [6] In addition to point mutations within *APP*, duplication of the *APP* locus on chromosome 21 has also been identified in families with familial early-onset AD and CAA. [15] Functional studies have clarified that these mutations within *APP* result in altered β-amyloid biological properties and subsequent deposition, much like in sporadic CAA.

Motivated by the close phenotypic and biological overlap between sporadic and familial CAA, we investigated whether any of the established rare variants or novel variants in the same protein region of *APP* might contribute to susceptibility to sporadic CAA.

## Methods

### Ethics Statement

The Institutional Review Board of the Massachusetts General Hospital approved all aspects of this study, and written informed consent for participation in the present study was obtained from all participating subjects or their surrogates.

### Patients

Subjects consisted of patients referred to Massachusetts General Hospital over an eleven year period who qualified for the diagnosis of *probable* or *definite* CAA according to the Boston Criteria. [4] Application of these criteria allows for reliable diagnosis of CAA in living subjects in the absence of cortical biopsy specimens, using clinical and neuroimaging data to assign patients to different groups, based on the likelihood of CAA pathology being present (Table 1).

**Table 1:** Boston criteria for CAA diagnosis.

**1. Definite CAA**
Full postmortem examination demonstrating:
• Lobar, cortical, or corticosubcortical hemorrhage
• Severe CAA with vasculopathy*
• Absence of other diagnostic lesion
**2. Probable CAA with supporting pathology**
Clinical data and pathologic tissue (evacuated hematoma or cortical biopsy) demonstrating:
• Lobar, cortical, or corticosubcortical hemorrhage
• Some degree of CAA in specimen
• Absence of other diagnostic lesion
**3. Probable CAA**
Clinical data and MRI or CT demonstrating:
• Multiple hemorrhages restricted to lobar, cortical, or corticosubcortical regions(cerebellar hemorrhage allowed)
• Age ≥55 years
• Absence of other cause of hemorrhage‡
**4. Possible CAA**
Clinical data and MRI or CT demonstrating:
• Single lobar, cortical, or corticosubcortical hemorrhage
• Age ≥55 years
• Absence of other cause of hemorrhage‡

*As defined in reference 20.

‡ Other causes of intracerebral hemorrhage include:

• excessive warfarin dosing (INR >3.0)

• antecedent head trauma or ischemic stroke

• CNS tumor

• vascular malformation

• CNS vasculitis

• blood dyscrasia

• coagulopathy.

Note: INR.3.0 or other nonspecific laboratory abnormalities permitted for diagnosis of possible CAA.

### Sequencing of APP Exons 16 & 17

Exons 16 and 17 of *APP* were amplified using a standard polymerase chain reaction (PCR) protocol. SNP detection was performed automatically with the SNP Compare analysis suite (Broad Institute of MIT and Harvard), which collates and compares SNP calls from two SNP calling algorithms, PolyDHAN and PolyPhred. [16] The quality of a given SNP is scored based on its detectability in each sequencing direction (forward and reverse) as determined by each SNP calling algorithm, as well as its estimated false positive rate. SNP Compare estimates false positive rates for detected variants using data derived from the International HapMap Consortium's ENCODE resequencing efforts. [17]

### Copy Number Assessment

We used multiplex ligation-dependent probe amplification (MLPA) (MRC Holland, Amsterdam, Holland) to assess for the presence of duplication of *APP.* Genomic DNA from ten unaffected individuals and one individual with trisomy-21 were included as negative and positive controls, respectively. Four synthetic paired probes for *APP* exons and four paired probes specific for genes flanking the *APP* locus were designed. Additional MLPA probes targeting sequences on chromosomes other than chromosome 21 were included as internal controls. All *APP* paired probe ligation sites were located in the coding regions of the gene. Probes for regions flanking *APP* were designed to detect duplications in genes previously found to show copy number variation in individuals with autosomal-dominant early-onset Alzheimer Disease with CAA. [15]

### Statistical Analyses

Novel variants and/or duplication of the *APP* locus in sequenced patients were used to infer the probability of identical findings in the general population of sporadic CAA patients by computing exact confidence limits for binomial proportions based on available sample size [18]. Findings from our study were compared to the theoretical binomial distribution (any *APP* mutation vs. no APP mutation) in the general population the study subjects were sampled from, i.e. sporadic CAA patients diagnosed according to the Boston criteria.

## Results

Of the 58 subjects (Table 2), 35 met criteria for probable CAA on radiographic and clinical criteria alone, while CAA was pathologically confirmed in 23 individuals (6 from autopsy reports and 17 from pathology reports from biopsy-obtained tissue). MRI data for CAA diagnosis was available for 32 (55%) subjects. Of all enrolled subjects, 13 (22%) had a single ICH before the event qualifying them for enrollment, while 2 (2%) had an history of multiple intracerebral bleeds. Five patients (9%) were being treated with warfarin at time of diagnosis. Family history of ICH and dementia was present in 3 (5%) and 2 (3%) individuals respectively.

**Table 2:** Characteristics of Patients with Sporadic CAA.

	Copy Number	Exon 16	Exon 17
Total Number of Patients	36	55	58
**Diagnosis** (%)			
Definite CAA (autopsy report)	4 (11)	6 (10)	6 (10)
Probable CAA (supportive biopsy pathology)	9 (25)	14 (25)	17 (29)
Probable CAA (supportive neuroimaging data)	23 (64)	35 (64)	35 (61)
**Age at Diagnosis** (Mean ± SD)	72±9	73±9	73±8
**Female** (%)	17 (47)	28 (51)	32 (55)
**Race/Ethnicity** (%)			
White	35 (97)	51 (92)	55 (94)
African American	1 (3)	2 (4)	1 (2)
Asian	0 (0)	1 (2)	1 (2)
Hispanic	0 (0)	1 (2)	1 (2)
**Family History of intracerebral hemorrhage** (%)	2 (5)	2 (4)	3 (5)
**Family History of Dementia** (%)	5 (14)	2 (4)	2 (3)

Sequencing of exon 16 was successful in 55 patients while MLPA was completed in 36. No novel variants or known mutations were identified in exons 16 and 17 and no duplication of the *APP* locus or in the four investigated flanking genes (*NCAM2, C21orf42*, *CYYR*1, *USP16*) was detected. Based on available sample size, exact confidence limits for binomial proportions estimate the frequency of mutations investigated in the present study as ranging from 0% to 8% in individuals with probable CAA in the general population.

## Discussion

We found no evidence that novel variants or established causal mutations for familial CAA have a role in sporadic CAA. These data suggest that rare mutations responsible for familial CAA are of such potency in their effect that they are unlikely to be found in patients without the familial syndrome. Furthermore, our results indicate that if *APP* exons 16 and 17 indeed harbor additional CAA-causing rare variants, their limited expected frequency in the sporadic CAA population suggests the overall associated attributable risk is likely to be minimal.

Based on these conclusions systematic genetic screening of CAA patients lacking a strong family history of hemorrhage or dementia is not warranted at this time. Familial and sporadic CAA are unlikely to share individual risk mutations.

Our study has several limitations. Based on previous genetic findings and functional studies in familial CAA, we only examined sequenced variation in exons 16 and 17 of *APP*. It is possible that variants in other exons or, of course, non-coding regions of *APP*, may influence the risk of sporadic CAA. However, current insight into the metabolism and deposition of β-amyloid suggest that mutations in exons 16 and 17 are often associated with functional consequences in Aβ properties, something which has not been described for coding or non-coding variants outside of these exons. In addition, our sample size was limited by the number of individuals whose clinical evaluation allowed qualification for the diagnosis of probable CAA. While systematic autopsy studies demonstrate that CAA is a common pathological finding in the elderly, its demonstration during life generally requires MRI or biopsy, which limits the number of individuals whose eligibility for studies like ours. [19]–[21] However, selection of CAA patients based on manifestation with ICH is likely to enrich our study cohort with more severe cases, presumably increasing our chance to observe CAA-associated mutations.

Susceptibility to a vast number of complex diseases is determined at least in part by the cumulative contribution of multiple common DNA polymorphisms of small effect. Sequence variants with large effects, however, may also contribute to variation in complex traits, such as circulating lipid levels. [22] Our results do not exclude a role for genetic variation in *APP* as a risk factor for sporadic CAA. Indeed, the clinical and biological overlap between familial and sporadic CAA suggests that there will be areas of shared genetic risk. Nonetheless, further investigation of *APP* variants in sporadic CAA is likely to require substantially larger samples of both cases of sporadic CAA and unaffected controls, a scale requiring large-scale multi-center collaboration.

## References

[1] Vaxillaire M, Froguel P (2008). Monogenic diabetes in the young, pharmacogenetics and relevance to multifactorial forms of type 2 diabetes.. Endocr Rev.

[2] Gloyn AL, Weedon MN, Owen KR, Turner MJ, Knight BA (2003). Large scale association studies of variants in genes encoding the pancreatic β-cell K-ATP channel subunits Kir6.2 (KCNJ11) and SUR1 (ABCC8) confirm that the KCNJ11 E23K variant is associated with increased risk of type 2 diabetes.. Diabetes.

[3] Florez JC, Burtt N, de Bakker PI, Almgren P, Tuomi T (2004). Haplotype structure and genotype-phenotype correlations of the sulfonylurea receptor and the islet ATP-sensitive potassium channel gene region.. Diabetes.

[4] Knudsen KA, Rosand J, Karluk D, Greenberg SM (2001). Clinical diagnosis of cerebral amyloid angiopathy: Validation of the boston criteria.. Neurology.

[5] Chen YW, Gurol ME, Rosand J, Viswanathan A, Rakich SM (2006). Progression of white matter lesions and hemorrhages in cerebral amyloid angiopathy.. Neurology.

[6] Smith EE, Gurol ME, Eng JA, Engel CR, Nguyen TN (2004). White matter lesions, cognition, and recurrent hemorrhage in lobar intracerebral hemorrhage.. Neurology.

[7] Zhang-Nunes SX, Maat-Schieman ML, van Duinen SG, Roos RA, Frosch MP (2006). The cerebral beta-amyloid angiopathies: Hereditary and sporadic.. Brain Pathology.

[8] Woo D, Sauerbeck LR, Kissela BM, Khoury JC, Szaflarski JP (2002). Genetic and environmental risk factors for intracerebral hemorrhage: Preliminary results of a population-based study.. Stroke.

[9] Bakker E, van Broeckhoven C, Haan J, Voorhoeve E, van Hul W (1991). DNA diagnosis for hereditary cerebral hemorrhage with amyloidosis (dutch type).. Am J Hum Genet..

[10] Miravalle L, Tokuda T, Chiarle R, Giaccone G, Bugiani O (2000). Substitutions at codon 22 of alzheimer's abeta peptide induce diverse conformational changes and apoptotic effects in human cerebral endothelial cells.. J Biol Chem.

[11] Nilsberth C, Forsell C, Axelman K, Gustafsson C, Luthman J (1999). A novel app mutation (e693g). The arctic mutation, causing alzheimer's disease with vascular symptoms.. Soc Neurosci Abs.

[12] Grabowski TJ, Cho HS, Vonsattel JP, Rebeck GW, Greenberg SM (2001). Novel amyloid precursor protein mutation in an iowa family with dementia and severe cerebral amyloid angiopathy.. Ann Neurol.

[13] Hendriks L, van Duijn CM, Cras P, Cruts M, Van Hul W (1992). Presenile dementia and cerebral haemorrhage linked to a mutation at codon 692 of the beta-amyloid precursor protein gene.. Nature Genetics.

[14] Obici L, Demarchi A, de Rosa G, Bellotti V, Marciano S (2005). A novel abetapp mutation exclusively associated with cerebral amyloid angiopathy.. Ann Neurol.

[15] Rovelet-Lecrux A, Hannequin D, Raux G, Le Meur N, Laquerriere A (2006). App locus duplication causes autosomal dominant early-onset alzheimer disease with cerebral amyloid angiopathy.. Nature Genetics.

[16] Nickerson DA, Tobe VO, Taylor SL (1997). PolyPhred: automating the detection and genotyping of single nucleotide substitutions using fluorescence-based resequencing.. Nucleic Acids Res.

[17] The International HapMap Consortium (2005). A haplotype map of the human genome.. Nature.

[18] Rosner B (2000). Fundamental of Biostatistics..

[19] Greenberg SM, Vonsattel JP (1997). Diagnosis of cerebral amyloid angiopathy. Sensitivity and specificity of cortical biopsy.. Stroke.

[20] Vonsattel JP, Myers RH, Hedley-Whyte ET, Ropper AH, Bird ED (1991). Cerebral amyloid angiopathy without and with cerebral hemorrhages: A comparative histological study.. Ann Neurol.

[21] Fernando MS, Ince PG, MRC Cognitive Function and Ageing Neuropathology Study Group (2004). Vascular pathologies and cognition in a population-based cohort of elderly people.. J Neurol Sci.

[22] Cohen JC, Kiss RS, Pertsemlidis A, Marcel YL, McPherson R (2004). Multiple rare alleles contribute to low plasma levels of HDL cholesterol.. Science.

